# Assessment of clinical variables as predictive markers in the development and progression of colorectal cancer

**DOI:** 10.1080/21655979.2021.1933680

**Published:** 2021-06-06

**Authors:** Mahmood Rasool, Arif Malik, Sulayman Waquar, Qura Tul Ain, Rabia Rasool, Muhammad Asif, Nisreen Anfinan, Absarul Haque, Hina Alam, Sagheer Ahmed, Mohammad Hamid Hamdard

**Affiliations:** a Center of Excellence in Genomic Medicine Research, Faculty of Applied Medical Sciences, King Abdulaziz University, Jeddah, Saudi Arabia; b Department of Medical Laboratory Technology, Faculty of Applied Medical Sciences, King Abdulaziz University, Jeddah, Saudi Arabia; c Institute of Molecular Biology and Biotechnology, the University of Lahore, Lahore, Pakistan; d Department of Biotechnology, BUITEMS, Quetta, Pakistan; e ORIC, Buitems, Quetta, Pakistan; f Gynecology Oncology Unit, Obstetrics and Gynaecology Department, Faculty of Medicine, King Abdulaziz University Hospital, Jeddah, Saudi Arabia; g King Fahd Medical Research Center, King Abdulaziz University, Jeddah, Saudi Arabia; h Pakistan Institute of Medical Sciences, Islamabad, Pakistan; i Shifa College of Pharmaceutical Sciences, Shifa Tameer-e-Millat University Islamabad; j Faculty of Biology, Kabul University Afghanistan

**Keywords:** COX-2, inflammation, Malondialdehyde (MDA), tumor necrosis factor-alpha (TNF-a), isoprostanes, C-reactive protein

## Abstract

Colorectal cancer (CRC) is graded as one of the most common cancer. It accounts for the second leading cause of cancer deaths worldwide. The present study intends to investigate the role and importance of different biochemical variables in the development of colorectal cancer.

In this cross-sectional study we recruited ninety-one patients diagnosed with colorectal cancer and fifty-three age-sex matched controls from June 2017 to June 2018. Different variables *i.e*. SOD, GSH, CAT, MDA, TGF, VEGF, TNF, ILs, MMPs, etc., were estimated with the help of their respective methods. Our findings suggest a significant increase in the levels of different inflammatory and stress-related markers. The NFκB, TGF-β, VEGFβ, 8OHdG, IsoP-2α were significantly found to be increased in patients with colon cancer (0.945 ± 0.067 μg/ml, 18.59 ± 1.53 pg/ml, 99.35 ± 4.29 pg/ml, 21.26 ± 1.29 pg/ml, 102.25 ± 4.25 pg/ml) as compared to controls (0.124 ± 0.024 μg/ml, 8.26 ± 0.88 pg/ml, 49.58 ± 2.62 pg/ml, 0.93 ± 0.29 pg/ml, 19.65 ± 3.19 pg/ml). Notably, the levels of different antioxidants were shown to be significantly lower in patients of colon cancer. The present study concluded that excessive oxidative stress and lipid peroxidation result in a decrease in the antioxidative capacity of cells which may influence diverse signaling cascades including NF-KB, which results in DNA modification and gene transcription that ultimately involved in the progression of colon cancer.

## Graphical Abstract

**Figure:**
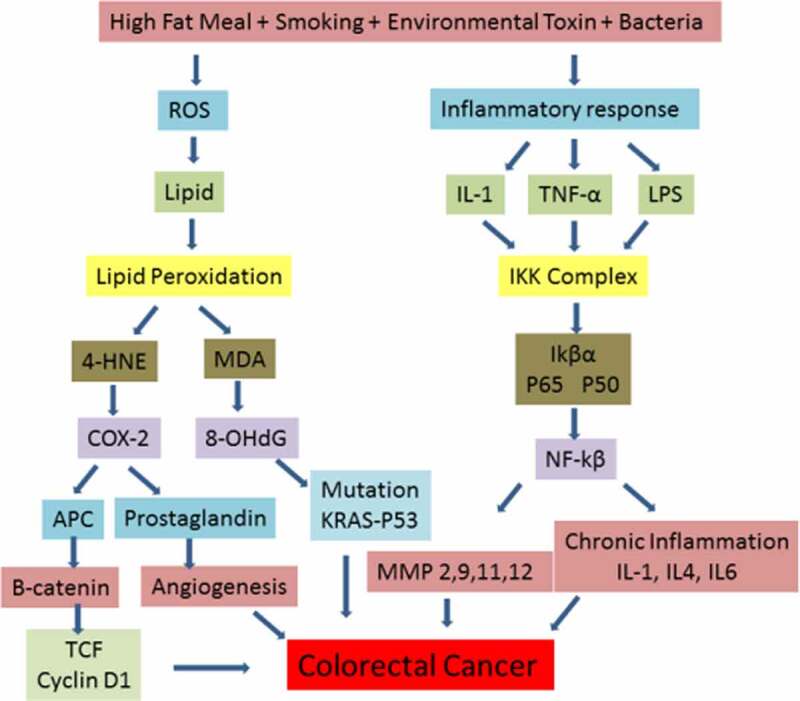

## Introduction

Colon cancer is one of the most common forms of human neoplastic diseases and is considered the most frequent cause of death, with 56,000 deaths and 140,000 new cancer cases worldwide [1,2]. Colon cancer begins from the intestinal epithelial cells lining the bowel. These epithelial cells with a high metabolic rate divide rapidly on the inner lining of the colon and outer growth in the form of polyps is formed. Adenomatous polyp, also called adenomas, is a precancerous stage that eventually leads to cancer and spread in later stages [1,3]. However, several pathways have been found to be involved in the pathogenesis of colon cancer. However, the three most common pathways include microsatellite instability, chromosomal instability, and the serrated adenocarcinoma pathway [4]. Notably, several risk factors have been identified that may trigger colon cancer such as physical inactivity, nutritional imbalance, alcohol consumption, and obesity as well as other environmental factors. The crosstalk between these risk factors and oxidative stress could potentially lead to the development and progression of colorectal cancer. Increased oxidative stress into the intestinal lumen and continuous exposure of intestinal mucosa to oxidative stress may cause significant damage to DNA that triggers the genetic mutation. The mutation in the gene responsible for DNA repair system and cell cycle may result in uncontrolled proliferation of cells thereby representing the initial mechanism involved in the carcinogenesis [5]. Notably, the human carcinomas display a significant elevation in the level of different stress markers such as tyrosine oxidation, isoprostane, MDA and NO formation, etc [6].

Patients suffering from inflammatory bowel disease (IBD) such as Crohn’s disease and ulcerative colitis may have an increased risk of colon cancer. The physiological imbalance in intestinal microbiota might be considered as a major risk factor in colon cancer development in ulcerative colitis patients [7]. The exposure of mucosal cells to inflammatory stimuli may also lead to an increased risk of colon cancer in ulcerative colitis. The disruption of gut microbiota in the intestine might cause persistent inflammatory reactions as demonstrated by inflammatory mediators both systemically and locally. Various types of inflammatory cells are infiltrated into colon tumors and cells involved in innate immune response such as natural killer cells (NK), mast cells, dendritic cells, specific macrophages associated with tumors and neutrophils can be detected easily in colorectal cancer patients. Moreover, the recruitment of cells of the adaptive immune response may also play a significant role in the colon and colitis-associated carcinomas that function as either pro or anti-tumorigenic activity. Receptors of different tumor-promoting cytokines associated with intestinal epithelial cells activate different transcription factors with their oncogenic responses such as NFκB, mTOR, and STAT3 which are particularly potential oncogenic factors involved in the development of colon cancer [8]. The tumor progression relies upon several components of the microenvironment such as various oxidants, pro and inflammatory cytokines, and extracellular matrix components. The activation of TNF-α, IL-6, IL8, and VEGF triggers downstream signaling involved in promoting tumor growth and invasiveness to other parts of the body [9]. The C reactive proteins (CRP) are produced in the liver as a result of the inflammatory response stimulated by several cytokines. The elevated level of CRP could be established as an important diagnostic biomarker marker for monitoring the development of colon cancer [10]. The role of matrix metalloproteinases (MMPs) is well recognized as an important factor in the remodeling process for normal growth and differentiation of tissues. It functions basically by the degradation of matrix proteoglycans and glycoproteins. The overexpression of several MMPs during carcinogenesis causes unregulated degradation of several extracellular matrix components resulting in distant metastasis. The expression levels of different MMPs during colon carcinomas may be correlated with advanced cancer stage and poor prognosis [11].

The present study was designed to estimate the levels of different inflammatory and stress-related markers to investigate the development and progression of colon cancer.

## Materials and methods

### Study design and subjects

In this study, we recruited 91 patients diagnosed with confirmed colorectal cancer and compared with 53 age-sex matched controls. The current study was conducted at the Institute of Molecular Biology and Biotechnology from June 2017 to June 2018 according to the ethical guidelines of the Institutional Review Board (IRB), the University of Lahore. Informed written consent was taken from all participants of the study according to Helsinki’s declaration.

### Selection criteria

For inclusion criteria; the subjects selected for the current study were confirmed cases of colorectal cancer. For exclusion criteria; The patients with type I diabetes mellitus, alcohol consumption, with known liver or with liver enzyme concentrations higher than three times the upper limit or any chronic infection such as tuberculosis, sarcoidosisetc., hemolytic anemia, hemoglobin variants were excluded from the current study.

### Blood sample collection

Five (5 ml) venous blood was drawn from each patient using specialized vacutainer. Serum was separated with the help of centrifugation and stored at −72°C for future analysis.

### Biochemical analysis

Levels of several serum markers were measured in the groups of patients and controls for their analysis. Serum levels of Malondialdehyde (MDA), Catalase (CAT), Superoxide dismutase (SOD) and Reduced glutathione (GSH) were determined in the study groups with the help of their respective spectrophotometric methods [12–15], Matrix metalloproteinases (MMPs) [16], Nitric Oxide (NO) [17], while other markers such as TNF-alpha, NFκB, VEGF-β, TGF-β, IL, COX-2, 8-OHdG, isoprostanes, Creatinine, Hs-CRP, TAS, and protein carbonyl were evaluated by the help of their respective ELISA kit protocols.

### Statistical analysis

For the statistical interpretation of the current study, the results of different parameters were determined for their significance using SPSS (v.16). All values were expressed as mean ± SD. P-value < 0.05 was considered to be statistically significant. Pearson linear correlation was used to study the correlations between different parameters.

## Results

The results of the current study show a significant increase in the levels of different stress and inflammatory markers and a decrease in the levels of different antioxidants in patients with colon cancer. The respective results of all parameters are represented in Table 1 and evidently shows that creatinine was significantly fluctuated (p = 0.011) as increased trends (2.95 ± 0.56 mg/L) were observed in colon cancer patients as compared to healthy individuals (0.78 ± 0.023 mg/L). Elevated levels of Hs-CRP (4.19 ± 1.58 mg/L) were most evident in patients suffering from colon cancer as compared with the controls (0.93 ± 0.014 mg/L). The p-value was found to be highly significant for [8-OHdG (p = 0.004) and isoprostranes (IsoP-2α) (p = 0.000)], respectively, and further both [8-OHdG = 21.26 ± 3.19 pg/ml and IsoP-2α = 102.25 ± 7.59] levels were recorded significantly higher in the colon cancer patients as compared to the healthy group. Further, a significantly increased level of MDA (7.89 ± 1.47 nmol/ml) was observed in colon cancer as compared to controls. The levels of COX-2 were found to be significantly increased (5.56 ± 1.18 ng/ml) (p = 0.024) in the patients group. The TNF-α levels in colon cancer subjects and controls were 41.56 ± 2.47 pg/ml and 24.29 ± 2.18 pg/ml, correspondingly. The highly significant (p-0.000) i.e. elevated raised levels of NF-Kβ were recorded in patients’ groups (0.945 ± 0.067 µg/ml) in contrast to normal group (0.124 ± 0.0014 µg/ml).

**Table 1.** Levels of biochemical markers in patients with colorectal cancer

**VARIABLES**	**CONTROL** **(n = 53)**	**PATIENTS** **(n = 91)**	**P-VALUE** **(p = <0.05)**
**CREATININE (mg/L)**	0.78 ± 0.099	2.95 ± 0.25	0.011
**Hs-CRP (mg/L)**	0.92 ± 0.12	4.19 ± 0.58	0.038
**8-OHdG (pg/ml)**	0.93 ± 0.29	21.26 ± 1.29	0.004
**TNF-α (pg/ml)**	24.29 ± 2.18	41.56 ± 2.47	0.031
**IsoP-2α (pg/ml)**	19.65 ± 3.19	102.25 ± 4.25	0.000
**MDA (nmol/ml)**	0.99 ± 0.14	7.89 ± 0.78	0.007
**COX-2 (ng/ml)**	0.74 ± 0.1	5.56 ± 0.65	0.024
**Total NFkB p65 (µg/ml)**	0.124 + 0.024	0.945 + 0.067	0.000
**SOD (IU/ml)**	1.16 ± 0.09	0.45 ± 0.041	0.009
**GSH (µmol/ml)**	7.89 ± 1.05	4.56 ± 0.68	0.006
**CAT (µmol/ml of Prot.)**	5.69 ± 0.51	3.15 ± 0.22	0.027
**NO (µmol/L)**	18.47 ± 2.17	47.59 ± 4.21	0.014
**MMP-9 (pg/ml)**	19.65 ± 5.02	84.26 ± 4.26	0.017
**MMP-11 (pg/ml)**	27.59 ± 2.21	51.26 ± 1.58	0.017
**MMP-2 (pg/ml)**	9.26 ± 1.44	19.58 ± 1.58	0.031
**MMP-19 (pg/ml)**	16.35 ± 2.31	46.35 ± 3.19	0.019
**VEGF-β (pg/ml)**	49.58 ± 2.62	99.35 ± 4.29	0.010
**Protein Carbonyl (µmol/ml)**	2.99 ± 0.38	9.68 ± 0.97	0.000
**TGF-β (pg/ml)**	8.26 ± 0.88	18.59 ± 1.53	0.021
**TAS (µmol/ml)**	21.59 ± 1.75	11.29 ± 0.99	0.000
**IL-6 (pg/ml)**	9.58 ± 1.12	28.26 ± 1.88	0.001
**IL-1 (pg/ml)**	6.35 ± 0.88	17.26 ± 1.77	0.011
**IL-4 (pg/ml)**	21.25 ± 1.99	32.18 ± 1.45	0.021
**IL-35 (pg/ml)**	0.174 ± 0.009	0.061 ± 0.007	0.031

The data presented in Table 1 show reduced antioxidant levels and thus clearly indicate that there is an increase in oxidative stress. The levels of enzymatic antioxidants such as SOD (0.45 ± 0.017IU/ml vs. 1.16 ± 0.84IU/ml), catalase (3.15 ± 0.058 µmol/ml vs. 5.69 ± 1.45 µmol/ml), and GSH (4.56 ± 1.48 µmol/ml vs. 7.89 ± 2.45 µmol/ml) were significantly declined in patients suffering from colon cancer as compared to normal subjects, respectively. Nitric oxide (NO) levels were significantly decreased in colon cancer patients’ group 47.59 ± 4.89 µmol/L as compared to control group 18.47 ± 2.17 µmol/L, correspondingly. The result presented in the tabular form of different matrix metalloproteinases in Table 1 depicts their vibrant effect in the progression of colon cancer. The levels of all MMPs including MMP-2, MMP-9, MMP-11, and MMP-19 were reported significantly high in patients with colon cancer [MMP-2 (19.58 ± 1.58 pg/ml), MMP-9 (84.26 ± 4.26 pg/ml), MMP-11 (51.26 ± 1.58), and MMP-19 (46.35 ± 4.19 pg/ml)] compared to their MMP profile [MMP-2 (9.26 ± 1.44 pg/ml), MMP-9 (19.65 ± 6.56 pg/ml), MMP-11 (27.59 ± 3.26), and MMP-19 (16.35 ± 3.33 pg/ml)] in normal individuals.

The data compiled in Table 1 suggest that serum VEGF-β levels of patients suffering from colon cancer were higher (99.35 ± 4.29) as compared with healthy individuals (49.58 ± 7.49) and thus the VEGF-β level shows statistically high significant (p = 0.010) between the two groups. Protein Carbonyl levels varied significantly as increased drifts (9.68 ± 1.49 µmol/ml) were experiential in colon cancer patients as compared to controls (2.99 ± 1.48 µmol/ml). The highly significant decreased levels of TAS (11.29 ± 1.48 µmol/ml) were evident in patients suffering from colon carcinoma as compared to healthy subjects (21.59 ± 2.59 µmol/ml). The significantly high serum levels of TGF-β in the patients were recorded as compared with the control group. The significant increase in the level of interleukin such as IL-1, IL-4, IL-6 was recorded in colon cancer patients (17.26 ± 2.49 pg/ml, 32.18 ± 1.45 pg/ml, 28.26 ± 1.88 pg/ml) as compared to controls (6.35 ± 1.07 pg/ml, 21.25 ± 2.18 pg/ml, 9.58 ± 3.33 pg/ml), respectively. Notably, the levels of IL-35 were found to be decreased and such a trend was observed in patients suffering from colon cancer (0.061 ± 0.028 pg/ml) as compared to that of control (0.174 ± 0.014 pg/ml) because of its anti-inflammatory properties.

## Discussion

Colon cancer is the third most common type of cancer and it accounts for approximately 0.6 million deaths per year and the mortality rate is increasing with each passing year [18]. Several endogenous and exogenous factors are known to be involved in the pathogenesis of colon cancer. The exogenous factors include lifestyles such as smoking, environmental pollutants, and obesity and other dietary factors that may cause a significant increase in the risk of colon cancer. The interplay between several genetic and environmental factors modulates the ability of epithelial cells lining of the gut to cope with damaging metabolic changes. The results of the current study demonstrate a significant increase in the level of oxidative stress markers in patients suffering from colon cancer. Epithelium of GI tract is continuously exposed to multiple stimuli of pro-oxidants from infections or other residents of microbiota or ingested food and gastric acids, etc. and as a result, these stimuli can cause extensive tissue damage which in turn triggers pathogenesis and progression of colon cancer [19]. Notably, one study in 2007 by Goyette *et al*. demonstrated a positive correlation between inflammatory bowel disease severity and oxidative stress. Overwhelming exposure of oxidants causes severe tissue restitution and ulceration inside the colon resulting in an increased risk of cancer [20]. Further, another study reported that increased production of oxygen radicals causes increased lipid peroxidation that results in the degeneration of cellular membrane and DNA damage in colon cancer [21]. Our results clearly suggest that the significant increase in the level of MDA, isoprostanes, and 8-OHdG in colon cancer patients is consistent with a previous study in 2005 conducted by Skrzdlewska *et al*. who clearly indicated the formation of lipid peroxidation products in colon cancer patients. Similarly, Ozturk *et al*. also reported similar results that showed a significant increase in the level of MDA both in circulation and as well as in tissue samples of colon cancer patients [22].

The increase in the production of lipid peroxidation products also damages the other cellular proteins resulting in the formation of protein carbonyl compounds in colon cancer patients and these findings are consistent with another study conducted by Yongsheng *et al*. in 2013 [23]. Superoxide anions and free radicals also cause the inactivation of several antioxidant enzymes catalase (CAT) and superoxide dismutase (SOD). The lowest activity of catalase enzyme was reported in tumor tissues. The catalase enzyme is used to defend against hydrogen peroxide generated by numerous reactions or by the action of enzyme superoxide dismutase. The significant decrease in the expression of SOD mRNA was reported in colon patients [24]. The present study also depicts an inverse correlation between MDA and SOD (MDA Vs. SOD, r = −0.661) as shown in Table 2. The current study clearly demonstrated increased level of C reactive protein (CRP) in the serum of colon cancer patients and these studies are consistent with several lines of evidence of other studies that indicated the increased risk of colon cancer in patients with chronic inflammatory response [25,26]. The IL-6 produced in chronic inflammation has been shown to stimulate the CRP production in colon cancer cell lines [27]. In this perspective, more elaborative studies on specific types of other cytokines are needed to establish reliable and robust specific inflammatory markers in order to fully interpret the inflammatory mechanism involved in colon carcinogenesis [9]. However, the 10-fold increased concentration of CRP level was reported in colorectal cancer patients as compared with healthy control by Tasilidis et al [28]. Notably, a significant correlation of CRP and IL-6 was demonstrated in the current findings (CRP Vs. IL-6, r = 0.741).

**Table 2.** Pearson s’ correlation coeeficients of different variables in patients with colorectal cancer

**VARIABLES**	r	**P(<0.05)**
IL-6 vs. VEGF-β	0.561	0.021
TNF vs. NF-Kb	0.648	0.000
IL-6 vs. Hs-CRP	0.741	0.003
MMP-9 vs. TGF-β	0.542	0.018
TNF-α vs. IL-35	−0.614	0.032
COX-2 vs. MMP-9	0.719	0.011
MDA vs. SOD	−0.661	0.017
COX2 vs. VEGF	0.489	0.000
CRP vs. creatinine	0.518	0.023

** Correlation is significant at the 0.05 level (Two-tailed)

One of the characteristic hallmarks of cancer progression is hypoxia-induced cytokines such as vascular endothelial growth factor (VEGF), TNF-a, interleukins, etc [29]. The tumor necrosis factor is the mediator of both acute and chronic inflammation. TNF-a, IL-6, and IL-8 are directly involved in tumor progression as well as indirectly through inductive VEGF expression [30–32]. Although, a wide variety of cytokines have been measured and evaluated in CRC tissue and blood but high circulating levels IL-6 and IL-8 have been correlated with advanced stages of disease with unfavorable prognosis [32,33]. Similarly, IL-6 and IL-8 have been correlated with advanced stages of disease with unfavorable prognosis in many cancer [34]. The IL-6 expression associated with tumor progression, invasiveness and resistance to chemotherapy in CRC cells have been also extensively reported [31,35]. Shiga et al. have shown that IL-6 levels were elevated in stage II CRC patients as compared with stage III patients [36]. For example, the IL-6 expression associated with tumor progression, invasiveness and resistance to chemotherapy in ovarian cancer cells have been also extensively reported [37]. Cohen et al. have demonstrated that IL6 inhibition in cisplatin induced overexpressing ovarian cancer cells resulted into significant sensitization to cisplatin, suggesting IL-6 involvement in the induction of platinum resistance [38]. Comparative study of IL-6 G/C SNPs at (−174) of, C allele also found to be potentially correlated with initial stage of tumor along with increased length of disease-free (DFS) and overall survival in both colorectal and ovarian cancer [39,40]. The IL-8 is also most thoroughly investigated cytokine, and an elevated serum and expression level has been correlated with advanced tumor stage, high tumor grade, and poor survival in many cancers including CRC [41,42]. Notably, the IL-8 mRNA as well as protein expression level were significantly found to be lower in non-metastatic and low grade metastatic CRC cell lines as compared with the high grade metastatic CRC cells, hence suggesting the potential metastatic link of IL8 in CRC (Aihua et al., 2001). Thus, IL-8 not only linked to promote malignant transformation [43], but also associated with inflammatory pathways and induction of gastric cancer [44]. Similarly, in ovarian cancer also IL-8 levels has been found to be up regulate instantly under exposure of chemotherapeutic agents [45]. Several studies have established that serum IL-8 is a promising biomarker for CRC detection and may become a clinically potential biomarker to identify high-risk patients [41,42]. Interestingly, the most functional SNPs in IL-8 promoter at position −251 bp has been associated with elevated plasma levels [46], as well as increase promoter activities [47]. Of note, this allele has also been correlated with an increased risk of developing many cancers including gastric, prostate and breast cancers [48].

**Figure 1.**
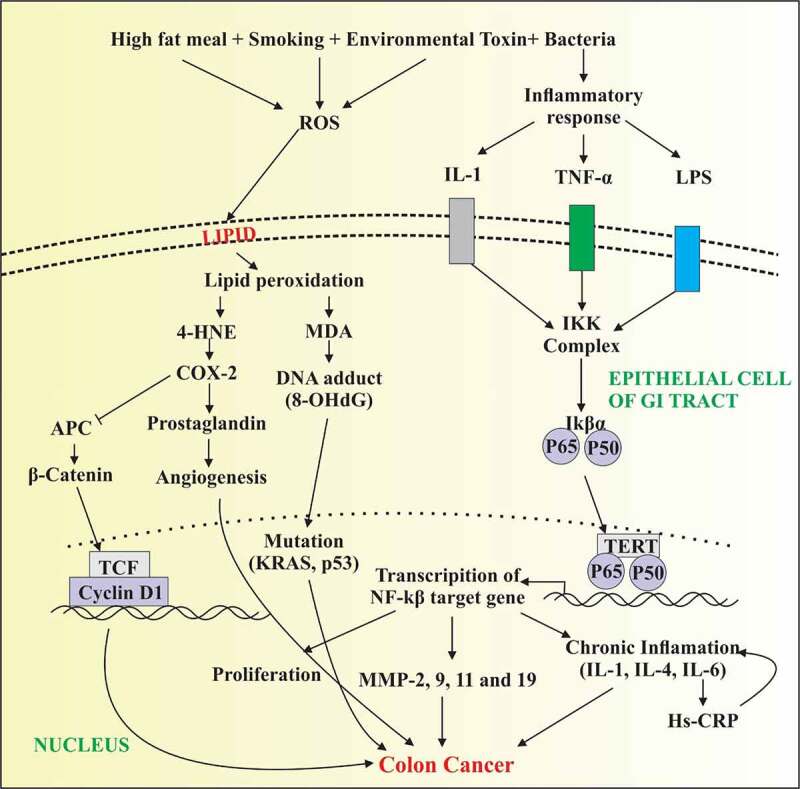
Depicting increased production of reactive oxygen species and inflammatory response that in turn trigger the activation of TNF-α, IL-1 and release of lipopolysaccharides (LPS) under external stimuli. Elevated level of free radicals cause damage to plasma membrane of epithelial cells of GI tract with increased formation of lipid peroxidation products. Further, 4-HNE activates COX2 production resulting in the activation of prostaglandin and B catenin that increase angiogenesis and proliferation of colon carcinomas. In parallel, LPS released from bacterial toxin and TNF- α cause the activation of NFkB signaling resulting in the transcription of NFkB target genes. Activation of several cytokines and MMPs cause increased proliferation of cancer cells resulting in tumor metastasis

Notably, our findings are in accordance with the results of the current study which shows elevated levels of TNF-a, IL-6, and VEGF in patients suffering from colon cancer. The IL-1 along with TNF-a, when secreted by inflammatory cells, triggers the inflammatory cascade of several other pro-inflammatory markers including COX-2, chemokines, and matrix metalloproteinases [49]. Moreover, IL-1 intensifies the expression of integrins on endothelial cells, stromal cells, and leukocytes and thus endorses cell intrusion into inflamed tissues. The COX-2 is a mediator for the synthesis of prostaglandin and its expression is up-regulated in colon cancer [50]. The apoptotic inhibition by overexpression of Bcl2 through MAPK or PI3 kinase-AKT signaling cascades is included in the pro-tumorigenic mechanism of COX-2. The production of VEGF and FGF induced by COX-2 triggers tumor angiogenesis and augments the dissemination of the tumor by transforming the adhesive features of cells and enhancing MMP activity [51–53]. The current findings indicate a significant correlation of COX2 and MMP-9 (COX2 vs MMP-9, r = 0.719 and COX2 Vs VEGF r = 0.489) as shown in Figure 1. The current findings indicate the elevated level of COX2 in colon cancer patients in comparison with normal and these findings are consistent with the previous study of Eberhart *et al*., 1994 [53]. The COX2 over-expression is positively correlated with malignant and premalignant lesions in epithelial cells of the gastrointestinal tract. The higher expression of COX2 in tumor tissues indicates its aggressiveness. The study of Eberhart *et al*. showed a significant increase in the level of COX2 mRNA in colon carcinoma as compared with other adenomas [53]. Further, another study also showed an elevated level of COX2 in colon cancer in contrast to normal mucosa [54]. The current study demonstrated the unusual pattern of IL-35 up-regulation in terms of its expression as compared to other interleukins. The other interleukins such as IL-1, IL-4, and IL-6 have been found to be up-regulated while the IL-35 has been shown to be down-regulated in colon cancer patients and this observation is in accordance with another finding of Zhang et al., 2017. IL-35 exhibits anti-inflammatory and immune-suppressive properties. It diminishes the progression, migration of colon cancer via B-catenin inhibition both at mRNA and protein levels. Β-catenin is involved in colon cancer development [55] The IL-35 seems to augment colon cell apoptosis and decrease cancer stem cells [56]. Interestingly, the present study shows an inverse correlation of IL-35 with TNF-α (IL-35 Vs. TNF-α r = −0.614).

The matrix metalloproteinases are a huge zinc-endopeptidases family that is involved in the degradation of the extracellular matrix and it is an important process in development, proliferation, neoplastic or inflammatory processes [57]. The cooperated and coordinated work between integrins and matrix metalloproteinases are crucial for the cancer cell metastasis and extracellular matrix invasiveness [58,59]. The levels of MMPs including MMP-2, MMP-9, MMP-11, and MMP-19 are elevated in the colon cancer patients as compared to normal individuals. In one study, Kryczka *et al*. closely examined the interface of B1 integrins and MMP-2 in cells of colon cancer and revealed that MMP-2 is up-regulated in invasive colorectal cancer. MMP-2 is involved in the degradation of B1 integrins, thus increasing motility and diminishing adhesion of tumor cells [60]. Several signaling cascades play a part in gelatinases activation. The SMAD proteins are intricate in the signaling of TGF-B and involved in the regulation, differentiation, and apoptosis of the cell cycle. The Smad4 is attached to the receptor and thus regulated SMADs overwhelm colon cancer cell migration by regulating MMP-9 activity.

In colon cancer, p38 gamma MAPK overexpression was leading to enhanced c-Jun synthesis, which in turn triggers the amplification of the transcription of MMP-9 and MMP-9 dependent invasion [61]. Correspondingly, TGF-B receptor kinase inhibitors decrease the expression of MMP-9 and inhibit colorectal metastasis [10,62]. The current study demonstrates that MMPs including MMP-2, MMP-9, MMP-11, and MMP-19 levels are increased in patients suffering from colon cancer which is concurrent with the studies of Yang *et al*., 2014; Langenskiold *et al*., 2005, Xu and Xu, 2014, and Sena *et al*., 2012 [63–66]. Creatinine is the most conventional biomarker of renal functioning for cost-effective and most convenient diagnosis. The recent findings indicate a significant increase in the level of creatinine in colon cancer patients indicating renal dysfunction in colon cancer patients. The study of Nerpin *et al*. reported that the cytokine-mediated inflammation and elevated level of CRP are strongly associated with creatinine levels indicating inflammation-induced renal damage [66]. The significant correlation between CRP and creatinine was demonstrated in the current findings (CRP vs creatinine r = 0.518).

## Conclusion

From the results of the above findings, it is concluded that the interplay of oxidative stress and inflammation is likely to be an integral factor in the pathogenesis of colon cancer. Oxidative modification of protein and lipids in malignant cells and its spread to the other neighboring nonmalignant cells cause extensive damage to DNA. The complex network of intestinal cytokines promotes several key hallmarks of cancer with genetic instability, angiogenesis, metastasis, and invasiveness. Hence, targeting cytokine signaling and proper antioxidant supplementation directly within the metastatic niche may be an important therapeutic approach in order to prevent the life-threating progression of the disease.

## Highlights

1. Importance of different biochemical markers in the development of CRC.
2. The levels of different antioxidants are significantly lower in CRC patients.
3. The inflammatory and stress related markers are increased in CRC patients.
